# *Actinomyces* and Alimentary Tract Diseases: A Review of Its Biological Functions and Pathology

**DOI:** 10.1155/2018/3820215

**Published:** 2018-08-26

**Authors:** Jun Li, Ying Li, Yu Zhou, Changzheng Wang, Benyan Wu, Jun Wan

**Affiliations:** ^1^Department of Gastroenterology, General Hospital of PLA, No. 28 Fuxing Road, Haidian District, Beijing, 100853, China; ^2^Department of Oncology, General Hospital of PLA, No. 28 Fuxing Road, Haidian District, Beijing, 100853, China; ^3^Department of Nanlou Clinical Laboratory, General Hospital of PLA, No. 28 Fuxing Road, Haidian District, Beijing, 100853, China

## Abstract

*Actinomyces* are nonmotile, filamentous, Gram-positive bacteria that cause actinomycosis in immunodeficiency patients. Although the prognosis of actinomycosis is good, the diagnosis of actinomycosis is quite difficult. Recent studies on actinomycosis have shown that* Actinomyces* play an important role in various biological and clinical processes, such as the formation of dental plaque and the degradation of organics in the gastrointestinal tract. Here, the distribution of* Actinomyces* in the digestive tract, and different biological effects of actinomycosis, and its clinical association with inflammatory diseases are discussed. Furthermore, an overview of the most commonly used treatment methods and drugs used to treat* Actinomyces* infected alimentary canal diseases is presented.

## 1. Introduction

**Figure 1:**
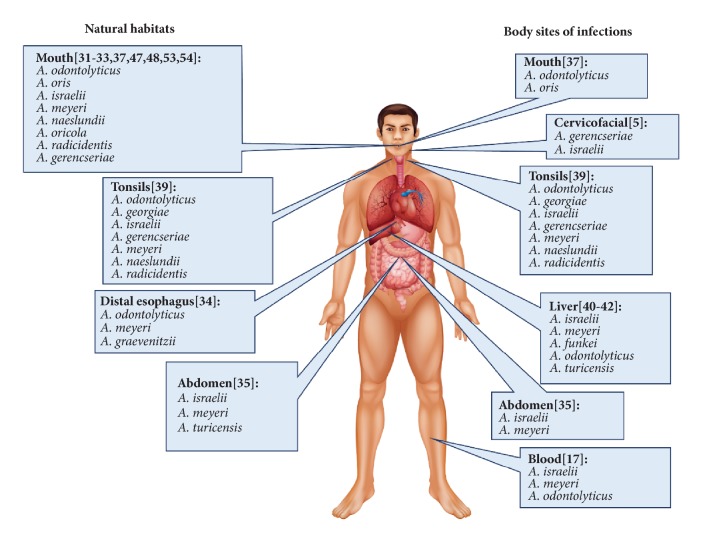
Natural habitats and infection sites of* Actinomyces* species.

*Actinomyces* species (*A.*) are nonmotile, filamentous, Gram-positive, and obligate anaerobic bacteria, which are facultatively pathogenic commensal inhabitants of the oral cavity, pharynx, gut, genitourinary tract, and skin [1].* Actinomyces* also readily cause actinomycosis in immunodeficiency patients, which is an indolent progressing granulomatous disease [2]. Actinomycosis can affect all tissues and organs, categorized as cervicofacial, thoracic, abdominopelvic and other types of actinomycosis [3] (Figure 1). Although the prognosis of these infections normally is good with medical and surgical treatments, actinomycosis still can lead to the death of patients due to the difficulties of early diagnosis and the severe infection diffusion of* Actinomyces* [4].

The mechanisms of pathogenicity of* Actinomyces* are not completely understood, but the invasion of breached or necrotic tissue has been proposed to be the initiating event for* Actinomyces* to penetrate and proliferate in deeper body structures [5]. Some studies have paid attention to* Actinomyces* related systemic infections including central nervous system (CNS) and cardiovascular and digestive tract diseases [6–9]. In the present review, we provide a brief review of (1) the distribution of* Actinomyces* species in the alimentary canal, (2) the biological function of* Actinomyces* species, (3) actinomycosis diseases in the digestive tract with diagnosis and treatment, and (4) possible correlations between the* Actinomyces* species and other inflammatory diseases of the alimentary tract.

## 2. Overview of* Actinomyces* Species and Actinomycosis

*Actinomyces* species, belonging to the phylum Actinobacteria, order Actinomycetales, and family Actinomycetaceae, are ubiquitous, occurring in soil and in the animal and human microbiota. Although currently a total of 47* Actinomyces* species have been identified, among which 25 are found in the human microbiota [10], advanced molecular techniques have been applied for taxonomic reassessment [11, 12] and revealed novel* Actinomyces* genera [13, 14]. The* Actinomyces* phylum includes the pathogens* Corynebacterium*,* Propionibacterium*,* Mycobacterium*, and* Nocardia*. In addition,* Propionibacterium propionicum* and* Bifidobacterium dentium* have been documented as important pathogens involved in infections with similar symptoms to actinomycosis [15, 16]. However,* A. gerencseriae and A. israelii* are the most commonly reported species found in human forms of the disease, which are responsible for about 70% of orocervicofacial infections [5], whereas* A. israelii*,* A. meyeri*, and* A. odontolyticus* are mainly associated with the rare hematogenous dissemination of actinomycosis [17]. It is noteworthy that most* Actinomyces* species are found colonizing polymicrobial flora. Human actinomycosis lesions usually consist of other aerobic and/or anaerobic species including* A. actinomycetemcomitans*,* Eikenella corrodens*,* Capnocytophaga, Fusobacteria*,* Bacteroides, Staphylococci*,* Streptococci*, and* Enterobacteriaceae* [15, 18]. Therefore, the isolation and subsequent identification of the infecting bacteria by culture and pathology are crucial for the diagnosis of actinomycosis and the choice of adjunctive antibiotics due to its polymicrobial nature.

*Actinomyces* species are the causative agents for actinomycosis, which is a rare invasive bacterial disease and usually characterized by the formation of granulomatous tissue, necrosis and major reactive fibrosis, draining sinuses, abscesses, and the development of fistulas as the infection progresses [19]. Multiple different clinical features of actinomycosis at various anatomical sites have been described, which cervicofacial actinomycosis being the most commonly reported form, accounting for about 50% of all cases [20], followed by abdominal actinomycosis (20%) and thoracic actinomycosis (15-20%) [21]. Involvement of the CNS, bone, and skin are rare conditions, most likely occurring as a result of hematogenous spread or direct dissemination of the original infected lesions [22, 23]. It is widely assumed that the decline in the incidence of all forms of actinomycosis in recent years is due to improved oral hygiene and the organism's susceptibility to many antimicrobial drugs used for bacteria diseases. However, actinomycosis is still widely distributed worldwide and affects mostly middle-aged individuals, especially males [19, 24].

## 3. *Actinomyces* Species as an Important Member of the Microbiome in the Human Alimentary Tract

It should be noted that the bacterial microbiome in the human alimentary tract is associated with both health and disease. Bacteria contribute to the development of mucosal barrier functions and suppress the establishment of pathogens [25], but can also be a chronic inflammatory stimulus to adjacent tissues [26, 27]. As a type of human commensal flora that normally colonizes the oral mucosa and nasopharyngeal, gastrointestinal tract and urogenital tracts,* Actinomyces* species play an important role in human health [28]. Before the further analysis of diseases caused by* Actinomyces*, it is perhaps useful to discuss the microbiome of* Actinomyces* in different locations of the alimentary tract.

The Human Microbiome Project Consortium investigated the dominant bacteria present in the oral cavity including* Streptococcus, Haemophilus, Actinomyces*, and* Prevotella [29]*. Various* Actinomyces* species have been shown to emerge in the oral cavity in one-third of infants at the age of 2 months and the diversity of* Actinomyces* increases with age [30]. A recent human oral microbiome study showed that the main members of the human oral microbiota included* A. odontolyticus, A. gerencseriae, A. israelii, A. meyeri, A. naeslundii, A. oricola, A. radicidentis*, and others not yet identified [31].* A. odontolyticus* has been shown to be one of the most prevalent* Actinomyces* species and participates in the formation of the biofilm on teeth at all ages [32]. The involvement of* Actinomyces* species in the early stage of biofilm formation on teeth include* A. naeslundii, A. oris*, and* A. gerencseriae *[33]. However, not only in the oral cavity, species such as* A. odontolyticus, A. meyeri*, and* A. graevenitzii *were recently isolated as part of a stable environment with other bacteria in the distal esophagus [34] and* A. israelii*,* A. meyeri*, and* A. turicensis* were detected in the abdomen [35]. It is remarkable that* Actinomyces* species were detected as colonizers of the infant gastrointestinal tract by measuring the diversity of 16S ribosomal DNA (rDNA) in infant fecal samples using polymerase chain reaction (PCR) methodology [36].

The proportion of some* Actinomyces* species such as* A. odontolyticus* and* A. oris* differs between healthy individuals and patients with periodontitis [37], and some* Actinomyces* species including* A. turicensis, A. odontolyticus, A. israelii*, and* A. radingae* were isolated from the tongue surface and may be involved in producing oral malodor [38]. In addition, a recent study characterized the composition and diversity of* Actinomyces* species in tonsillar crypts and found that* A. odontolyticus* colonized both healthy subjects and tonsillitis patients suggesting that other oral* Actinomyces* species found in tonsillar crypts microbiota such as* A. georgiae, A. israelii, A. gerencseriae, A. meyeri, A. naeslundii*, and* A. radicidentis* may be involved in causing the disease [39]. For various types of actinomycosis in the alimentary tract, the major* Actinomyces* species leading to abdominal actinomycosis include* A. israelii* and* A. meyeri*. Actinomycosis involvement of the liver and biliary tract is rare but have been described [35]. In addition to* A. israelii* and* A. meyeri*, other* Actinomyces* species such as* A. funkei, A. odontolyticus*, and* A. turicensis* have been implicated in liver actinomycosis [40–42].

## 4. Biological Functions of* Actinomyces* Species

As one of the normal bacteria colonizing the digestive tract,* Actinomyces* species are not able to release exotoxins and their cellular components are not known to be toxic [43]. Formate, acetate, succinate, lactate, and various antibiotics are produced by the* Actinomyces* species, and the metabolic potential for* Actinomyces* might be to break down and recycle organic compounds in the human gastrointestinal system [28]. The specific pathogenesis of actinomycosis has still not been fully elucidated, but this type of infection is accompanied by long-term inflammatory lesions containing massive numbers of polymorphonuclear leukocytes (PMNs), macrophages, and plasma cells which are able to damage tissues by releasing hydrolytic enzymes [44].

Engel et al. suggested that the* A. viscosus* might release a chemotactic factor that is mainly responsible for the accumulation of PMNs and mononuclear cells;* Actinomyces* substances may have a direct effect upon monocytes once they arrive at the infected sites. Nevertheless, plasma cells may arise as a result of stimulation of B lymphocytes by specific* A. viscosus* antigens [45]. However, usually the* Actinomyces* species would not produce infections alone and actinomycosis often involves companion bacteria such as Gram-negative bacilli and anaerobic* Streptococci*. Jordan et al. established experimental actinomycotic infections in mice and the histological evidence showed that polymorphonuclear leukocytes were not able to penetrate and invade the developing central bacterial granule of* A. israelii* lesions, which resulted in the “unreachable attack” of the leukocytes towards the bacteria inside the granule, while the existence of the inside bacteria would enhance the viability of* Actinomyces* cells by producing an anaerobic environment. Thus, the companion bacteria would be able to elaborate the toxins and enzymes as well as inhibiting host defenses to facilitate infections [46].

Certain* Actinomyces* species in the oral cavity are developing the formation of a biofilm.* Actinomyces oris* is the predominant organism among many* Actinomyces* species known to colonize the human oral cavity in all age groups [47, 48]. It is able to express fimbriae to adherence saliva deposits on enamel and interbacterial associations [49, 50]. It is noteworthy that studies on the metabolism of* Actinomyces* species have revealed that the production of energy by most* Actinomyces* species is by glycolysis. This is true for healthy and carious root sites but different enzymes are involved [51], which indicated that the genus' prevalence may be related to sugar availability and that altered metabolism of carbohydrates in the* Actinomyces* species is able to cause dysbiosis in the biofilm. In addition, the organic acid produced by the metabolism of* Actinomyces* would lead to the accumulation of intracellular polysaccharides, representing a cariogenic trait in these bacteria [52]. A recent study pointed out that* A. naeslundii* can utilize urea as a nitrogen source to protect itself from environmental acidification inside the oral cavity and then would be a kind of superior bacteria against the nonureolytic organisms in dental plaque, acting as a determinant of plaque ecology [53, 54].

## 5. Actinomycosis in the Alimentary Tract after Diagnosis and Treatment

As analyzed previously, Actinomycosis happening in alimentary tract is quite rare, and from the studies of case reports presented in recent years it is not surprising to find that most of the patients were middle-aged or above with hypoimmunity. These kinds of diseases are usually combined with other infections, malignant tumors, and damage to the gastrointestinal mucosa [55–60].

More examples of actinomycosis in the alimentary tract accompanied with other diseases are presented. Lee et al. reported a 41-year-old man with a late stage HIV infection [59]. Meanwhile, a 30-year-old man with a previous endodontic treatment history contracted periapical actinomycosis [61], and a 27-yar-old male who had a history of renal transplantation for renal disease secondary to lupus was diagnosed with esophageal actinomycosis [62]. Also, Al-Obaidy et al. reported a case about primary gastric actinomycosis. Although the patient (87 years old) did not have a history of trauma or underwent abdominal surgery, the patient was prescribed medication over a long period for dyslipidemia, renal impairment, premature ventricular contractions, nonischemic cardiomyopathy, and hypertension. The author suggested that the drugs the patient took would have caused damage to the gastric mucosa and that age-related mucosal atrophy was able to induce diminished mucosal resistance [58]. Therefore, actinomycosis frequently occurs in immunodeficient or immunocompromised patients due to other infections or the prescribed medication.

The colon, cecum, and appendix are the gastrointestinal tract regions commonly associated with actinomycosis [29].* Actinomyces* species infection can proceed over many weeks or even years after destruction of the gastrointestinal mucosa. Previous surgical procedures for colonic diverticulitis with perforation or appendicitis are recognized predisposing factors [63]. Peitsidis et al. reported a 35-year-old woman who was diagnosed with appendix actinomycosis. This young woman had a long-standing intrauterine device, which can be a risk factor for infectious diseases [64].

Although the prognosis of infectious disease is generally good, there was still one case of actinomycosis in the mediastinum leading to the death of the patient. In this case, the infection spreads from the digestive tract to the mediastinum, finally leading to death. But it is noteworthy that this patient was initially very weak due to a long time infection and was therefore the patient not able to receive surgical treatment to debride the infected tissues. Therefore, physicians should always pay attention and raise high awareness about possible actinomycetic infections, when an early diagnosis of actinomycosis could significantly improve the clinical outcome [55].

*Diagnosis. *Any abscesses or persistent infections in the alimentary tract should be suggestive of actinomycosis, although the symptomatic diagnoses of these infectious diseases are often painless [65] and accompanied by other systemic diseases [58, 61, 62]. It is important for physicians to be aware that actinomycosis frequently ‘imitates' malignancy nocardiosis or tuberculosis [5] as it can spread to distant tissues and organs in the human body progressively to form abscesses, which is analogous to tumor metastasis through invasive mechanisms. The culture of* Actinomyces* is quite difficult because* Actinomyces* infections are likely to be polymicrobial as a result of previous antibiotic therapies and therefore more time is needed to culture* Actinomyces* in an anaerobic environment. On the contrary, inflammation caused by* Actinomyces* has significant characteristic features. The presence of “sulfur granules” at the infection sites is a typical histopathological change, which often contains abscesses with yellowish sulfur-like granules [66]. Thus, histopathological examination of infected tissue is generally a more sensitive technique compared to bacterial culture, possibly revealing typical yellowish sulfur granules containing filamentous Gram-positive bacilli and inflammatory cells [67].

For suspected actinomycosis in the alimentary tract, endoscopic examinations and CT scans are useful to gain an overview of the lesions. A biopsy should also be used to facilitate a histological examination [58, 59, 62], but, in some cases, actinomycotic infections can be identified after the histological examinations of surgical specimens, as the lesions are resected after misdiagnosis [55, 57]. In a rare case of ascending colon actinomycosis, Filippou et al. applied CT imaging to identify a tumor-like mass. The characteristics of the lesion and the diffuse inflammation of the mesentery suggested it might be a perforated ascending colon tumor, and then surgery was applied to this patient. After the microscopic examination of the surgical specimen, actinomycotic “sulfur granules” were detected [68]. Similarly, Lee et al. applied CT imaging and positron emission tomography to identify a small mass at the origin of the appendix in a 50-year-old woman. All the empirical evidence indicated it should be an appendiceal tumor but the histological examination of the surgical specimen revealed it was actinomycosis of the appendix [56].

Nucleic acid probes and PCR techniques have recently been developed to identify actinomycosis in an accurate and rapid manner. Hansen et al. applied a PCR method with mild decalcification to detect* A. israelii *in bone specimens and found that the detection sensitivity of these genera was remarkably improved. Therefore, improved molecular methods for the diagnosis of actinomycosis would be one option.

*For the Treatment. Actinomyces* do not produce beta-lactamases and often are susceptible to beta-lactams antibiotics notably amoxicillin and penicillin G. The effects of other broad-spectrum antibiotics against* Actinomyces*, such as cephalosporins, ceftriaxone, and piperacillin, remain controversial and the acquisition of resistant flora should be avoided [21, 69, 70]. At the same time,* Actinomyces* seems not to be sensitive to first-generation cephalosporins. Clavulanic acid, a beta-lactam inhibitor, should be combined to treat actinomycosis if copathogens, e.g.,* Enterobacteriaceae,* have been implicated in the infection [21, 69, 70]. Applying prolonged therapies with high doses of antibiotics is the key to cure actinomycosis as the induration of the infection sites, which means the blood supply would be insufficient for antibiotics to penetrate into the infected tissues. Although prolonged antimicrobial therapy is effective for most patients with actinomycosis, surgery should be considered in complicated cases. For example, patients with widespread necrotic tissues or who are nonresponsive to antibiotic therapy need surgical approaches to drain abscesses and relieve obstructions. In addition, for patients who underwent surgery, antibiotic therapy should be prolonged to prevent the recurrence of the infection [71, 72].

For actinomycosis in the oral cavity, Thukral et al. treated a male patient aged 35 years with intravenous penicillin and metronidazole followed by orally administered antibiotics for between 2 and 4 weeks [73]. For cases of esophageal actinomycosis, systemic intravenous penicillin treatment is usually needed, and the combination of penicillin G and amoxicillin has also been reported [60]. In one patient with gastric actinomycosis, the empiric antibiotic therapy of IV metronidazole and IV levofloxacin were prescribed resulting in a complete cure [58].

## 6. Possible Relationships between* Actinomyces* Species and Various Alimentary Tract Diseases

### 6.1. Periodontal Diseases

Periodontal disease is a type of inflammatory disease caused by biofilm that induces harm to the tooth-supporting tissues. Untreated periodontal disease is able to cause the loosening of teeth, bleeding of the gums and even the loss of teeth [74, 75]. The etiology and pathogenesis of periodontal disease remain unclear but it is believed to have connections with changes in the composition of the microbiota in subgingival tissues and also genetic factors [76]. A recent metagenomic analysis revealed that* Prevotella* is the most abundant species in periodontal plaque samples, followed by* Streptococcus*, Corynebacterium, and* Actinomyces* [77]. Patients with periodontal disease were often found to have overexpression of* Bacteroidetes* and* Porphyromonas gingivali*s and a reduced expression of* Treponema denticola* and* Actinomyces* [78], suggesting that changes of formation and interactions among microbiota members contributed to periodontal disease. However, further analysis is needed to investigate the association of the heterogenous microbiome with periodontal disease.

Although the occurrence of periodontitis seems not to be related to the* Actinomyces* species in the oral cavity, as one of the residential microbial communities, the alterations of the proportion of* Actinomyces* would change the community structure and subsequently change the subgingival ecologies [79]. In addition, Ye et al. revealed that the antibodies induced by* Streptococci* and* Actinomyces* could contribute to the progression of periodontitis and perturbation of the epithelial attachment to teeth [80]. Takeuchi et al. also indicated that the products of* Actinomyces* and the specific immune reactions caused by* Actinomyces* are able to cause damage to periodontal tissues [81].

### 6.2. Inflammatory Bowel Disease

Alterations of microbiota are believed to activate immune responses and contribute to inflammatory bowel disease (IBD), with Crohn's disease and ulcerative colitis (UC) being the most common types. UC causes inflammation of large intestine, while Crohn's disease can affect all components of the gastrointestinal tract including the mouth, esophagus, stomach, and small and large intestines. Recent studies on the molecular pathogenesis of IBD have revealed that people with susceptible genes are more likely to have a deficient epithelial barrier function and lack innate and adaptive immunities. [82, 83] Furthermore, commensal bacteria might be the driver of IBD rather than conventional pathogens [84].

The gut microbiome in patients with UC and Crohn's disease were found to be different from those found in a healthy population and between these two types of IBD as well. Although microbial biodiversity was reduced 30-50% in patients with IBD, overexpression of certain microorganism such as* Proteobacteria* and* Actinobacteria* was detected in UC and* Enterococcus faecium* and* Proteobacteria *in Crohn's disease. Current studies have revealed that* Actinomyces* species are not related to the pathogenesis of inflammatory bowel disease, but the changes in the enteric environment and immune factors caused by* Actinomyces* may aggravate the injuries caused by inflammation [85, 86] Furthermore, Takahashi et al. found that genera* Actinomyces* and* Bifidobacterium* increased significantly in pediatric Crohn's disease patients as determined from fecal samples analyzed by 16s rRNA sequencing [87]. Lewis et al. focused on the microbial dynamics when treating patients with Crohn's disease. They explored the independent effect of inflammation on the composition of the gut microbiota after therapy and reported that the abundance of* Actinomyces* was decreased [88].

### 6.3. Celiac Disease

Celiac disease (CD) is distinguished by intestinal inflammation induced by gluten, proteins found in the normal diet [89]. As a type of autoimmune disease, it is believed that there are certain connections between infections and CD. Holyces et al. indicated that* Actinomyces* species might be one of the risk factors for the development of CD because* A. graevenitzii* had an increased abundance in the small intestine of CD patients [90]. However, Fernandez-Feo et al. pointed out that gluten-degrading microorganisms in the upper gastrointestinal tract are able to cleave the toxic gluten fragments, which may give us a novel therapeutic method for the treatment of CD. They also confirmed that* A. odontolyticus* in the oral cavity had the ability to degrade gluten, which means its gluten-degrading enzymes could be a potential adjunctive therapy for CD patients [91].

## 7. Further Investigations of the Functions of* Actinomyces* in Alimentary Canal Diseases Are Required

Actinomycosis in the alimentary tract is rarely seen and the pathogenesis has not been fully studied. According to the retrieved literature, there are no relevant studies about the biological function of the* Actinomyces* species in the human digestive tract. There is a hypothesis that* Actinomyces* in the oral cavity would distribute into the esophagus, hepatobiliary, and gastrointestinal tracts accompanied with other pathogenic bacteria to cause infections following poor oral hygiene and oral mucosa trauma [67]. But some studies proved that several* Actinomyces* species colonized within the infant intestine as normal organisms so that normal* Actinomyces* species are also able to cause infections [30, 36]. In addition, complex biological interactions among these diverse bacteria and within the digestive tract greatly affect the healthy or disease status of the host [92].

Some cases and studies in animals indicated that the damage of the wall of the digestive tract was one of the leading etiology causes of actinomycosis [93], but primary gastric actinomycosis was also reported [58]. Therefore, further studies on the pathogenesis of the actinomycosis in the alimentary tract are required.

## 8. Conclusions

Actinomycosis caused by* Actinomyces *species in the alimentary tract is rare as well as satisfactory prognosis in the case of early diagnosis.* Actinomyces *species are distributed widely as part of the microbiome in the alimentary tract from the oral to intestinal tract and have different biological functions and clinical features of actinomycosis. Changes of formation and interactions of* Actinomyces *species with other microbiota members contribute to various alimentary tract diseases such as periodontal disease, IBD, and CD. Further analysis is needed to investigate the mechanisms of pathogenicity of* Actinomyces* in the context of microbiota in alimentary tract diseases. In addition to bacterial culture and pathological examination, molecular biological techniques including PCR and 16S RNA sequencing have recently been developed for the speedy diagnosis of actinomycosis and to identify accurately the infective* Actinomyces *species. Patients with actinomycosis may “mimic” other malignancy processes in different locations of the alimentary tract and usually require prolonged antibiotic therapy with or without surgery.
